# From Plan to Impact

**DOI:** 10.1002/alz70860_110732

**Published:** 2025-12-23

**Authors:** Dale Goldhawk

**Affiliations:** ^1^ Alzheimer's Disease International, Lincolnshire, IL, USA

## Abstract

The Global Action Plan will only have an impact on the lives of people with dementia and their caregivers if countries take meaningful steps to address the crisis, including by developing national dementia plans. Each year, Alzheimer's Disease International (ADI) tracks progress on the Plan's seven action areas. This presentation will review the latest analysis.

